# Del dolor en los jóvenes al dolor persistente: Una trayectoria evitable

**DOI:** 10.23938/ASSN.1117

**Published:** 2025-12-30

**Authors:** Ignacio Yurss Arruga

**Affiliations:** Servicio Navarro de Salud-Osasunbidea Berriozar Navarra España

La presencia en este número 48(3) de la revista *Anales del Sistema Sanitario de Navarra* del artículo titulado *Dolor musculoesquelético y calidad de vida relacionada con la salud en estudiantes universitarios españoles de ciencias de la salud*^1^, nos acerca al fenómeno del dolor aplicado a un segmento de la población que no es usualmente aludido. Este artículo de Beatriz Rodríguez-Romero y colaboradores resulta de gran interés, pues este grupo de edad empieza a relacionarse con la complejidad del mundo de la salud y puede ser un vector eficaz en el planteamiento de nuevos abordajes que ya se apuntan sobre este tema.

El dolor osteomuscular en los jóvenes puede entenderse no solo como una manifestación física, sino también como un fenómeno de carácter adaptativo frente al contexto de estrés que caracteriza a la juventud actual. En una sociedad marcada por la presión académica, las exigencias sociales y el uso intensivo de dispositivos electrónicos, el cuerpo responde mediante señales de alerta que buscan proteger y regular el equilibrio interno^2^. El dolor, en este sentido, actúa como un mecanismo adaptativo que obliga al joven a detenerse, modificar hábitos y reconocer los límites de su organismo, y que refleja la necesidad de aliviar la carga emocional y física.

Además, el carácter adaptativo del dolor osteomuscular se relaciona con la resiliencia juvenil. Al enfrentar estas molestias, los jóvenes desarrollan conciencia sobre la importancia de la actividad física moderada, la higiene postural y la gestión del estrés. De esta manera, el dolor no solo limita, sino que también educa y orienta hacia prácticas más saludables. Reconocerlo como un aliado en la prevención de daños mayores permite resignificar su impacto.

En los jóvenes, este fenómeno vinculado al estrés contemporáneo no debe interpretarse únicamente como un obstáculo, sino como una respuesta adaptativa que busca preservar la integridad física y emocional. Comprenderlo en este marco abre la posibilidad de transformar la experiencia dolorosa en una oportunidad de aprendizaje y crecimiento personal.

Es especialmente congruente con la realidad y pertinente en sus conclusiones cómo el dolor musculoesquelético y el malestar emocional constituyen dos de los problemas de salud más frecuentes en la mujer joven universitaria^1^. Diversos estudios han demostrado que el género influye en la mayor prevalencia de síntomas físicos y psicológicos en comparación con sus pares masculinos, lo que repercute directamente en su calidad de vida. La combinación de exigencias académicas, presiones sociales y cambios propios de la juventud en la mujer genera un escenario de vulnerabilidad que se traduce en dolor crónico, ansiedad y disminución del bienestar general. Analizar esta problemática resulta esencial para diseñar estrategias de prevención y apoyo dentro del ámbito universitario.

Estos aspectos no son ajenos a la problemática actual y al elevado impacto del dolor crónico, fenómeno no solo sanitario sino social, que se ha convertido en uno de los desafíos de salud más complejos y extendidos de la vida moderna. Se estima que afecta a millones de personas en todo el mundo, trascendiendo edades, contextos culturales y estilos de vida. Más allá de la sintomatología física, el dolor crónico impacta profundamente en la esfera emocional, social y profesional de quienes lo padecen, generando un círculo vicioso de limitaciones y sufrimiento que a menudo resulta difícil de romper. En este contexto, la comprensión del dolor como fenómeno multidimensional ha impulsado el desarrollo de nuevas estrategias terapéuticas más integrales, humanas y basadas en evidencia.

A diferencia del dolor agudo, que cumple una función protectora como señal de alerta ante una lesión o enfermedad -como indica Arturo Goicoechea, autor de *El dolor crónico no es para siempre*^3^ y de *Sapiens, ma non tropo*^4^-, el dolor crónico persiste más allá del tiempo habitual de curación -generalmente más de tres meses- y puede mantener su presencia incluso en ausencia de un daño físico detectable. Su naturaleza compleja ha sido ampliamente estudiada desde la neurociencia, la psicología y la medicina, dando lugar al concepto de *dolor como experiencia*, en el que intervienen factores biológicos, cognitivos, emocionales y sociales.

En la vida actual, el dolor crónico se ve potenciado por elementos característicos de la modernidad: el sedentarismo creciente, el estrés continuo, la sobreexposición a pantallas^5^, los ritmos laborales acelerados y la longevidad prolongada que incrementa la incidencia de enfermedades degenerativas. Todo ello crea un caldo de cultivo en el que el dolor persistente se vuelve un acompañante indeseable para muchas personas.

Actualmente emergen nuevas variables causales sobre el dolor crónico, como el dolor como adaptador relacional y vivencial, o la ganancia secundaria^6^.

Más novedosos todavía parece el enfoque que lanza la doctora Suzanne O’Sullivan en su libro *La era del diagnóstico,* en el que critica los abordajes *pro diagnóstico* de la medicina actual que condicionan comportamientos e, incluso, inducen a cambios de vida por los que hay pacientes que pasan de una calidad de vida normalizada a una vivencia como enfermos que lleva a discapacidad, sufrimiento, incertidumbre y demandas legales^7^.

Además, el impacto del dolor va más allá del malestar corporal: afecta la calidad del sueño, limita la movilidad, reduce la productividad laboral y puede desencadenar trastornos del ánimo, como ansiedad o depresión. Esta interacción constante entre cuerpo y mente dificulta el tratamiento cuando se emplean únicamente enfoques biomédicos, lo que explica por qué la comunidad científica y sanitaria ha ido adoptando modelos más integrales.

Durante décadas, el tratamiento del dolor se ha centrado principalmente en la prescripción de medicamentos analgésicos, desde antiinflamatorios no esteroideos hasta opioides en casos más graves^8^. Sin embargo, estos enfoques farmacológicos presentan limitaciones significativas: efectos secundarios adversos, riesgos de dependencia -especialmente con los opioides- y alivios temporales que no modifican las causas subyacentes del dolor.

Por otra parte, las actuaciones invasivas (bloqueos caudales, infiltración facetaria) e incluso quirúrgicas no siempre ofrecen soluciones definitivas y pueden generar complicaciones. Esto ha motivado una búsqueda intensa de alternativas que aborden el dolor de manera más segura, sostenible y centrada en el paciente.

En los últimos años, el modelo biopsicosocial se ha consolidado como el marco más aceptado para comprender y tratar el dolor crónico. Este enfoque reconoce que la experiencia del dolor está influida por tres tipos de factores: biológicos (inflamación, lesiones, condiciones médicas, genética), psicológicos (pensamientos catastrofistas, creencias sobre el dolor, manejo del estrés) y sociales (apoyo familiar, entorno laboral, acceso a cuidados de salud).

Adoptar este modelo, implica diseñar tratamientos individualizados que combinen intervenciones físicas, psicológicas y sociales^9^ Es precisamente dentro de este marco donde han surgido estrategias emergentes con resultados prometedores cuya descripción excede los límites de este editorial.

No obstante, me detendré brevemente en las terapias mente-cuerpo que integran ejercicios y técnicas de regulación emocional^10^^,^^11^; para ser más concretos, *mindfulness*^12^, yoga terapéutico^13^, tai chi, *qi gong* y *biofeedback* tienen el respaldo de la evidencia científica sobre su eficacia^14^. Aunque estos tratamientos requieren más investigación y regulación, representan un horizonte innovador en el abordaje del dolor musculoesquelético. Estas aproximaciones terapéuticas no sustituyen la atención médica, pero complementan eficazmente otros tratamientos y abordan la dimensión emocional del dolor.

Asimismo, nuevos enfoques conectados con la emergencia de las llamadas *Nuevas Tecnologías*, como los modelos de terapia digital y salud conectada, han permitido generar aplicaciones y plataformas de terapia que permiten un seguimiento personalizado de síntomas, programas de ejercicios guiados por fisioterapeutas virtuales, recordatorios de hábitos saludables, terapias cognitivo-conductuales en formato digital, o sensores *wearables* que monitorizan el movimiento o la postura. Estos modelos pueden democratizar el acceso al tratamiento y facilitan la adherencia a planes de autocuidado, lo que resulta fundamental en el manejo del dolor crónico.

La Atención Primaria de Salud puede jugar un papel crucial en estos planteamientos por su accesibilidad, multidisciplinariedad, trabajo en equipo y, muy especialmente, tras la incorporación de nuevos perfiles especializados en Psicología y Fisioterapia. No obstante, debe actualizar su modelo, adaptarlo a estos problemas emergentes y enfocarse más hacia los problemas de salud y menos al de dispensario asistencial más centrado en la demanda.

El desafío del dolor crónico no radica únicamente en su complejidad fisiológica, sino también en la necesidad de transformar el modo en que se acompaña al paciente. El futuro apunta, como hemos dicho, hacia tratamientos más integrales que no solo busquen eliminar el dolor, sino mejorar la calidad de vida y promover la autonomía.

Los enfoques emergentes, por tanto, reflejan un cambio de paradigma donde la ciencia, la tecnología y la empatía se entrelazan para ofrecer alternativas más seguras, sostenibles y adaptadas a las necesidades reales de las personas. El objetivo final ya no es simplemente silenciar el dolor, sino comprenderlo, abordarlo desde múltiples ángulos y acompañar al paciente en un proceso profundo de recuperación física y emocional.

Como refleja Vinay Prasad, hematólogo, *la medicina occidental es un sistema incompleto y fragmentado*^15^. Es momento pues, a través de este fenómeno sanitario de tan alto impacto, de acercarnos al abandonado Humanismo Médico como aproximación a los que sufren, algo que demandan con verdadera ansia nuestros pacientes con dolor y seguramente los jóvenes en particular.
